# High-Throughput Phenotypic Assay to Screen for Anthelmintic Activity on *Haemonchus contortus*

**DOI:** 10.3390/ph14070616

**Published:** 2021-06-26

**Authors:** Aya C. Taki, Joseph J. Byrne, Tao Wang, Brad E. Sleebs, Nghi Nguyen, Ross S. Hall, Pasi K. Korhonen, Bill C.H. Chang, Paul Jackson, Abdul Jabbar, Robin B. Gasser

**Affiliations:** 1 Department of Veterinary Biosciences, Melbourne Veterinary School, Faculty of Veterinary and Agricultural Sciences, The University of Melbourne, Parkville, VIC 3010, Australia; aya.taki@unimelb.edu.au (A.C.T.); byrnej1@unimelb.edu.au (J.J.B.); tao.wang1@unimelb.edu.au (T.W.); sleebs@wehi.edu.au (B.E.S.); rossh@unimelb.edu.au (R.S.H.); pasi.korhonen@unimelb.edu.au (P.K.K.); bchang@yourgene.com.tw (B.C.H.C.); jabbara@unimelb.edu.au (A.J.); 2 Chemical Biology Division, Walter and Eliza Hall Institute of Medical Research, Parkville, VIC 3052, Australia; nguyen.n@wehi.com.au; 3 Faculty of Medicine, Dentistry and Health Sciences, The University of Melbourne, Parkville, VIC 3010, Australia; 4 Johnson & Johnson, Global Public Health, Janssen Research and Development, San Diego, CA 92121, USA; PJackso3@its.jnj.com

**Keywords:** high throughput screening (HTS), phenotypic assay, infrared light-interference, motility, parasitic nematode, *Haemonchus contortus*

## Abstract

Parasitic worms cause very significant diseases in animals and humans worldwide, and their control is critical to enhance health, well-being and productivity. Due to widespread drug resistance in many parasitic worms of animals globally, there is a major, continuing demand for the discovery and development of anthelmintic drugs for use to control these worms. Here, we established a practical, cost-effective and semi-automated high throughput screening (HTS) assay, which relies on the measurement of motility of larvae of the barber’s pole worm (*Haemonchus contortus*) using infrared light-interference. Using this assay, we screened 80,500 small molecules and achieved a hit rate of 0.05%. We identified three small molecules that reproducibly inhibited larval motility and/or development (IC_50_ values of ~4 to 41 µM). Future work will critically assess the potential of selected hits as candidates for subsequent optimisation or repurposing against parasitic nematodes. This HTS assay has a major advantage over most previous assays in that it achieves a ≥ 10-times higher throughput (i.e., 10,000 compounds per week), and is thus suited to the screening of libraries of tens of thousands to hundreds of thousands of compounds for subsequent hit-to-lead optimisation or effective repurposing and development. The current assay should be adaptable to many socioeconomically important parasitic nematodes, including those that cause neglected tropical diseases (NTDs). This aspect is of relevance, given the goals of the World Health Organization (WHO) Roadmap for NTDs 2021–2030, to develop more effective drugs and drug combinations to improve patient outcomes and circumvent the ineffectiveness of some current anthelmintic drugs and possible drug resistance.

## 1. Introduction

Substantial socioeconomic benefits are gained from the chemotherapeutic control of parasitic worms of animals and humans. However, numerous challenges need to be overcome in a continued effort to discover new compounds with enhanced performance (safety, efficacy and convenience), particularly given the ineffectiveness of some treatments and the widespread drug resistance in many parasites of many host species [1,2]. Although substantial efforts have gone into vaccine discovery and development, there is currently no licensed, safe and effective vaccine against a human helminthiasis, and only a very small number of vaccines have been approved for use in animals in some countries [3,4,5,6,7,8], with Barbervax^®®^ against haemonchosis in livestock being one relatively recent example [7]. While vaccination (immunoprophylaxis) would be the preferred option for prevention, vaccines against gastrointestinal worms may not achieve sufficiently high levels of protection for long enough periods to sustain the prevention/control of disease, such that strategic anthelmintic treatment or integrated strategies will still be required [9]. Thus, there is a clear need for continued efforts to discover and develop new and effective anthelmintic compounds to achieve effective control.

Numerous reviews have discussed the challenges associated with anthelmintic discovery and development (e.g., [1,10,11]). In an academic context, a key constraint or ‘bottleneck’ has been a lack of quantitative, automated platforms for the phenotypic screening of large numbers of compounds or small molecules for their effects on whole parasites [12]. Clearly, the development of automated systems using the free-living nematode *Caenorhabditi*s *elegans* as a target organism has accelerated anthelmintic drug discovery, and advances in workflows, imaging platforms and computer algorithms have made this ‘model organism’ a useful option for high throughput screening (HTS) [1,12,13,14]. As one of the best characterised multicellular (metazoan) organisms, *C. elegans* is an excellent target organism for drug discovery, and extensive biological, genomic, transcriptomic, proteomic and functional genomic resources, tools and databases (particularly WormBase) enable such discovery efforts. Although *C. elegans* is not a parasite, it is related to a wide range of nematodes, including those of the order Strongylida (‘strongylids’; including species of *Haemonchus, Ostertagia, Teladorsagia, Trichostrongylus, Cooperia, Nematodirus*; *Ancylostoma, Bunostomum*; *Oesophagostomum, Chabertia*), which are of major animal or human health importance worldwide. Underpinned by extensive information and ‘infrastructure’ for *C. elegans*, there has been significant, recent progress in creating genomic and molecular resources for some of these parasitic nematodes, particularly for *H. contortus*—the ‘barber’s pole worm’ of ruminants—for which animal models are well-established for detailed biological and pharmacological studies [10,15,16].

*H. contortus* infects predominantly small ruminants (including sheep and goats) and causes haemonchosis; it lives in the stomach (abomasum), feeds on blood, causing anaemia and associated complications, leading to serious production losses and death in severely affected animals. *H. contortus* infection is transmitted orally via contaminated pasture to the host in a direct life cycle [17]: eggs are passed in host faeces on to pasture and, under favourable conditions, embryonate; within one day, eggs hatch and release individual first-stage larvae (L1s), which develop to third-stage larvae (L3) within approximately one week; infective L3s are then ingested by the host, exsheath (xL3) and, after a histotrophic phase, develop through fourth-stage larvae (L4s) to dioecious adults (within 3 weeks) in the abomasum.

Significantly complementing knowledge of the biology of *H. contortus* is the recent completion of a representative genome [18] and the availability of extensive molecular and biological resources for this species [15,19], which signals an elevation of the ‘status’ of this species to a genuine ‘model organism’ to represent the Strongylida and to underpin drug discovery work [10]. While, in a commercial context, advances in drug discovery and HTS platforms for *H. contortus* and related nematode species already exist, such technology, associated protocols and information are, obviously, not accessible by the broader scientific community. Thus, in an academic context, there has been a need to develop practical, low-cost screening platforms to assist drug discovery efforts, together with commercial and philanthropic partners in public-private partnerships (PPPs) [20].

Most conventional in vitro-screening methods for parasitic worms have had limitations in that most of them are time-consuming to perform and/or lack repeatability/reproducibility [1,12,21,22]. Egg hatch- and larval development-assays are laborious, have low reproducibility and use free-living rather than parasitic stages (the target of anthelmintic treatment). Phenotypic assays that measure a reduction in motility include the larval paralysis test [23] and larval migration assay [24,25], which are labour-intensive and rely on the visual/microscopic scoring of phenotypes or enumeration [21]. Motility measurement, based on object-tracking, has been applied to worms such as the blood fluke *Schistosoma mansoni* [26]. However, this approach, which relies on the digital segmentation of objects from the ‘background’ [22], can sometimes be challenging to apply to parasitic worms, depending on their movement characteristics and tendency to clump. Measuring motility via electrical impedance [27] or with infrared light beam-interference [28,29] shows considerable promise for developing medium to high throughput techniques.

In 2015, we established a practical and inexpensive phenotypic assay (96-well format) for the in vitro-screening of compounds for activity against larval stages of *H. contortus* [30]. Since that time, this assay was used to screen 15,860 synthetic compounds as well as 10,760 natural products and extracts thereof to complete numerous projects (reviewed in [11,31]). The assay, which relies on video/image capture via microscopy, has performed well and has had significant advantages over conventional methods in that it relies on the use of L3s—which can be stored for many months at constant temperature in an incubator, substantially reducing experimental animal use; it is well-suited for the screening of hundreds or thousands of compounds for hit discovery, subsequent hit-to-lead optimisation, repurposing and development; it provides phenotypic (morphological) information and should have applicability to other socioeconomically important parasitic worms. The throughput of this screening assay is ~1000 compounds per week. Despite the positive features of this assay [30], its throughput is too low for large-scale projects with industry or philanthropic collaborators, whose curated libraries often comprise tens or hundreds of thousands of synthetic compounds or natural product extracts. To overcome this limitation, we established here a practical, cost- and time-effective semi-automated HTS assay, which relies on the measurement of motility of *H. contortus* larvae using infrared light beam-interference as well as the subsequent assessment of developmental inhibition and phenotypic alterations.

## 2. Results

### 2.1. Selection of the Acquisition Algorithm in WMicrotracker ONE Instrument (Phylumtech, Argentina) for the HTS Assay

In the WMicroTracker ONE instrument, two acquisition algorithms (Mode 0_Threshold + Binary; Mode 1_Threshold Average; described in the user’s manual) were assessed for the optimal measurement of *H. contortus* xL3 motility by infrared light beam-interference in 384-well plates using a larval density of 80 xL3 per well. After a 90 h-incubation with the negative (LB* + 0.4% dimethyl sulfoxide, DMSO) and positive (monepantel) -controls, much lower activity counts (equating to motility) were captured using Mode 0 than Mode 1 in negative-control wells, resulting in Z’-factors and signal-to-background ratios of 0.48 and 1.5 for Mode 0 compared with 0.76 and 16.0 for Mode 1, respectively. Thus, Mode 1—which is a more quantitative algorithm—was selected for the acquisition of xL3 motility.

### 2.2. Optimisation of xL3s Density, Assessment of Physical Stimulation and Time for the Measurement of Motility in the Assay

**Figure 1:**
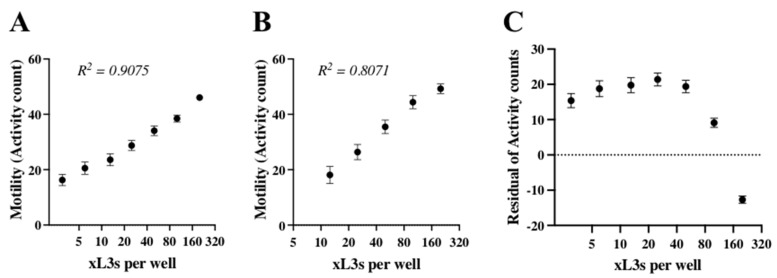
Optimisation of larval density for use in the screening assay. The correlation between the larval (xL3) motility and density using 384- (**A**) or 96-well (**B**) plates was determined by the Pearson’s correlation (R^2^) method after 90 h of incubation at 38 °C and 10% CO_2_. The residual plot from a linear regression analysis of larval motility in a 384-well plate using different numbers of xL3s per well (**C**); this plot shows a negative correlation for a larval density of > 100 xL3s per well. The larval density (xL3 per well) is log_2_-transformed for panels A to C. Data points represent 16 replicates; mean ± standard error of the mean (SEM).

First, experiments were conducted to establish the optimum density of xL3s in wells of plates for HTS (i.e., motility measurement). To do this, we undertook a regression analysis for motility (activity counts) using a two-fold serial dilution series (i.e., 3, 6, 12, 25, 50, 100 or 200 xL3s per well). We assessed and compared the correlation (R^2^) between larval density and motility using 96-well and 384-well plates. There was a clear relationship using both types of plates, with a higher coefficient (R^2^) achieved for the 384-well plate (91%) than the 96-well plate (81%) (Figure 1A,B). For the former data set (384-well plate), a residual analysis revealed a non-linear relationship between the larval density and motility, resulting in positive residuals for densities of up to 100 xL3s per well and in negative residuals for 200 xL3s per well, with variation (standard error of the mean, SEM) among replicates (*n* = 16) being less for densities of 50 xL3s and 100 xL3s (SEM: ≤ 1.7) than for ≤ 25 xL3s per well (SEM: ≥ 2.0) (Figure 1C). Based on these findings, we selected a density of 80 xL3s per well for the screening assay (in 384-well plates).

**Figure 2:**
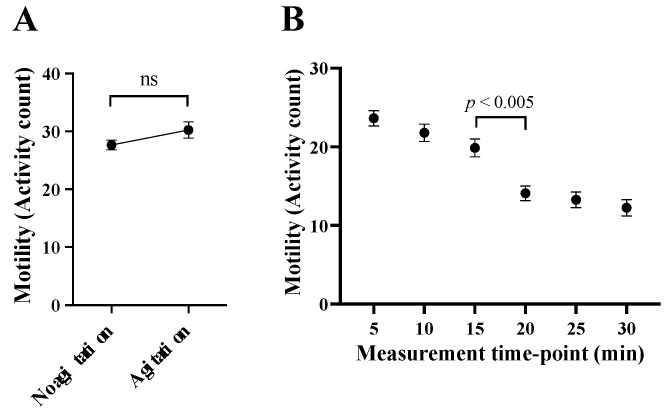
Evaluation of the effect of agitation and measurement time-point on larval motility. The effect of the agitation of 384-well plates—using an orbital shaker (120 rotations per min) for 15 min—on the larval motility (using 100 xL3s per well) was assessed after 90 h of incubation at 38 °C and 10% CO_2_ (**A**); no significant increase in larval motility was achieved by the agitation. The larval motility was observed over 30 min to determine the duration of measurement time by assessing the motility reduction over time (**B**). After 15 min, the motility decreased significantly; thus, the optimal duration for motility measurement was determined to be up to 15 min. Data points represent 60 replicates; mean ± standard error of the mean (SEM); ns = not significant.

Second, we assessed whether horizontal shaking (agitation) of the plate would have a significant stimulatory effect on the motility of xL3s just prior to measuring ‘activity counts’ in the WMicroTracker. This was undertaken to account for a possible effect of lethargus, known to occur during moulting/development [17]. After 90 h of in vitro-incubation, we showed that there was no significant difference in xL3 motility in 384-well plates (for 64 replicates), whether they were agitated for 15 min or not (Figure 2A). Third, we established when best to measure xL3 motility in 384-well plates (for 60 replicates). To do this, we measured motility every 5 min for 30 min, and showed that it decreased significantly at 20 min, compromising the signal-to-background ratio (Figure 2B). Based on these findings, we elected to measure motility (“activity counts”) for 15 min.

### 2.3. Establishing Compound Concentration, Hit-Identification Cut-off and IC_50_ Values for Reference Compounds

**Figure 3:**
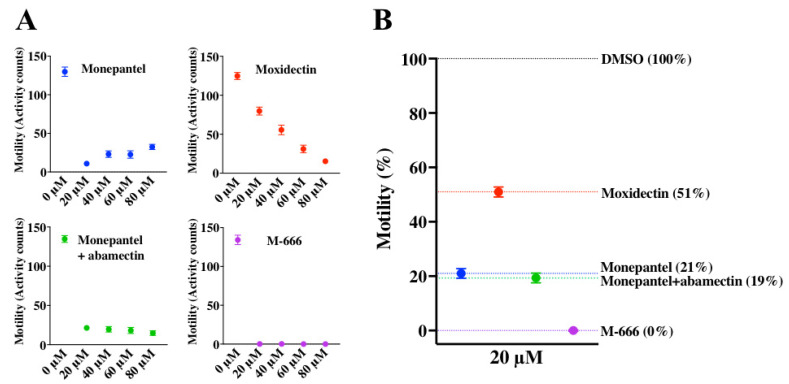
Evaluation of positive-control compounds in vitro against exsheathed third-stage larvae (xL3s) of *Haemonchus contortus*. Four positive-control compounds (monepantel, moxidectin, monepantel+abamectin and M-666) at concentrations of 20, 40, 60 and 80 µM were assessed in the 384-well plate assay for their effects on the larval motility. After 90 h of incubation, the motility (activity counts) was measured for 15 min (**A**) for each concentration, and the motility (normalised; %) of xL3s exposed to 20 µM of each of compounds was determined with reference to negative-control (0 µM, LB* + 0.4% DMSO). (**B**); monepantel and monepantel+abamectin each reduced larval motility by ~80%, and moxidectin reduced it by 50%, while M-666 completely abolished larval motility (100% inhibition). Data points represent 16 replicates; mean ± standard error of the mean (SEM).

We used compound M-666 [32] as a positive-control, and monepantel (Zolvix), monepantel+abamectin (Zolvix Plus) and moxidectin (Cydectin) as reference controls in our assay; monepantel has a different mode(s) of action to macrocyclic lactones including moxidectin [33,34], and M-666 is highly likely to have a unique mechanism of action [32]; the latter is the most potent compound. First, we established concentrations of control- and test-compounds for use in the assay. Positive-control compounds were assessed at 0, 20, 40, 60 and 80 µM (using 100 xL3s per well), and 20 µM was selected for routine testing/screening after 90 h (Figure 3A). The motility of xL3s exposed to test-compounds was compared with that in the negative-control wells (containing LB* + 0.4% DMSO) (Figure 3B). At 20 µM, after 90 h, M-666 (positive-control), monepantel + abamectin, monepantel and moxidectin (reference controls) achieved larval motility reductions of 100%, 81%, 79% and 49%, respectively. The threshold value for hit identification was set at ≥ 70% motility reduction, which compares with that set previously [30], although the assay allowed different empirical cut-offs to be set as/if required. Then, we established dose-response curves for compounds (via titration from 100 µM to 0.76 nM—two-fold serial dilution) in 96-well plates using a density of ~300 xL3s per well. IC_50_ values established for monepantel and moxidectin in this assay were 0.4 ± 0.09 µM and 0.08 ± 0.09 µM, respectively (Figure S1, Supplementary Materials).

### 2.4. Screening Results, Dose Responses and Phenotypes Detected

**Figure 4:**
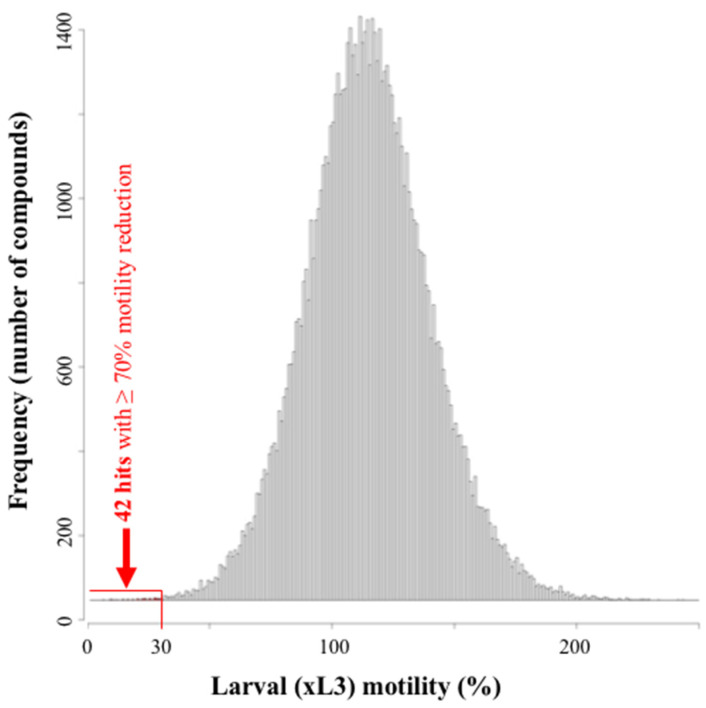
The distribution of the numbers of compounds according to the larval motility values obtained from the high throughput screen on third-stage larvae (xL3s) of *Haemonchus contortus.* Of the total number of 80,500 compounds individually tested at 20 µM, 42 of them (0.05%) reduced larval motility by ≥70% and were, thus, designated as ‘hits’. Motility values were normalised against those of positive (monepantel) and negative (no-compound) -controls.

Using the established assay, we screened the 80,500 compounds from the Jump-stARter library at a concentration of 20 µM. At 90 h, we identified 42 compounds that reduced xL3 motility by ≥70% (Table S1, Supplementary Materials), equating to an overall ‘hit rate’ of 0.05% (cf. Figure 4). At 168 h, seven of these 42 compounds induced detectable, abnormal (non-wildtype) larval phenotypes with reference to the wildtype (negative-control) (Table S1). In this primary screen, the main abnormal phenotypes detected were *Evisceration* (*Evi*), *Coiled* (*Coi*) and *Curved* (*Cur*), which were induced by five, one and one compounds, respectively (Tables S1 and S2, Supplementary Materials).

**Figure 5:**
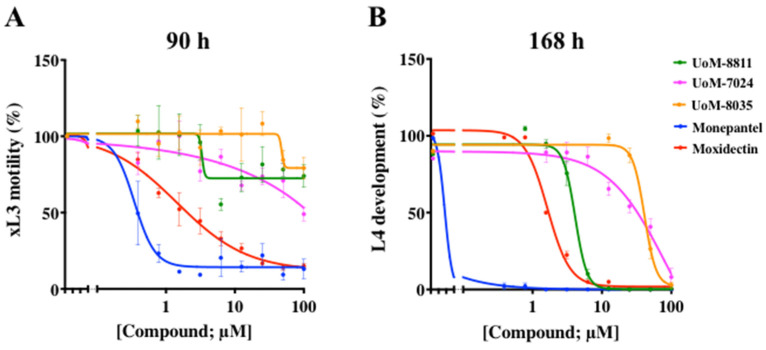
Potency of selected ‘hit’ compounds in vitro against exsheathed third-stage larvae (xL3s) and fourth-stage larvae (L4s) of *Haemonchus contortus*. Dose-response curves of the individual compounds (designated UoM-8811, UoM-7024 and UoM-8035) and each of two positive-control compounds (monepantel and moxidectin) assessed in 96-well plates [30] for inhibition of xL3 motility at 90 h (**A**), and for inhibition of larval (L4) development at 168 h (**B**). Each data point represents nine replicates; mean ± standard error of the mean (SEM) are indicated.

We selected three hit candidates (i.e., UoM-8811, UoM-7024 and UoM-8035) with xL3 motility reductions of between 72% and 91% at 90 h in the primary screen for further evaluation in the dose-response assay. At 168 h, UoM-8811, UoM-7024 and UoM-8035 markedly inhibited larval development in this assay, achieving IC_50_ values of ~4 µM, 25 µM and 41 µM, respectively (Figure 5), and induced abnormal phenotypes (*Cur* and *Evi*; Table S2, Supplementary Materials). A quantitative, temporal study of phenotypic changes induced from 90 h to 168 h in this dose-response assay showed that the *Cur* phenotype usually preceded *Evi* for each of these compounds. As there is no previous report of any of these three compounds having been tested against a parasitic nematode, current work is focused on their optimisation by medicinal chemistry, followed by subsequent structure–activity relationship (SAR), bioavailability and toxicity evaluations.

## 3. Discussion

**Figure 6:**
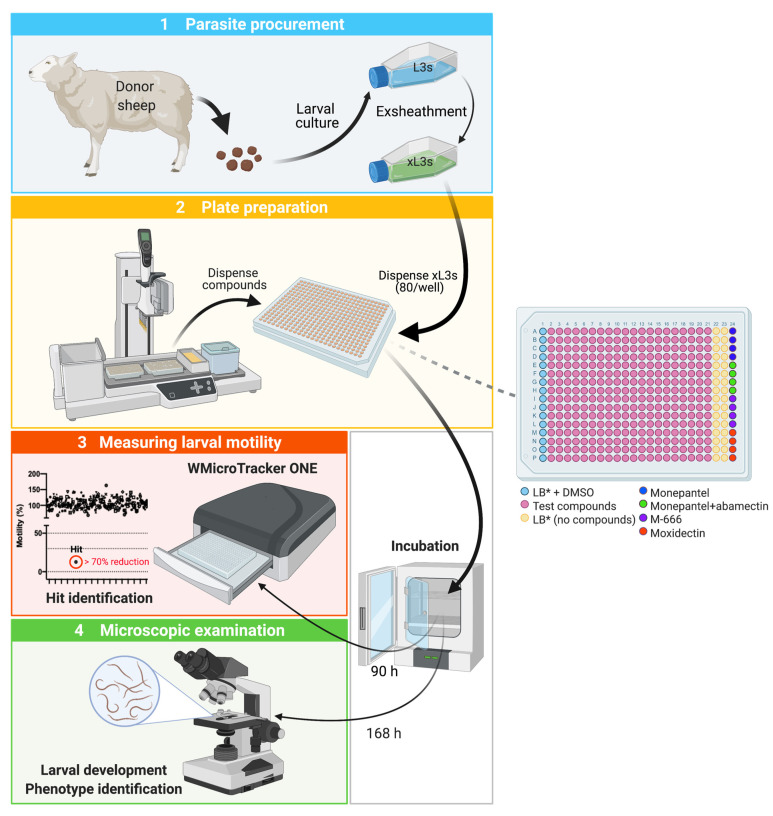
Workflow (four steps) used for the discovery of compounds with anthelmintic effects against larvae of *Haemonchus contortus*. ***Step 1—Parasite procurement:*** Donor sheep are experimentally infected with 7000–9000 *H. contortus* third-stage larvae (L3s) to produce a patent infection (after ~21–24 days). Faecal samples are collected from a sheep with a patent infection and then subjected to copro-culture at 27 °C and 100% humidity for one week to allow *H. contortus* eggs to embryonate, and larvae to hatch and develop; L3s are harvested, washed and then stored in sterile water at 11 °C (constant) for ≤6 months. Immediately prior to screening, L3s are exsheathed in 0.15% (*v*/*v*) sodium hypochlorite (NaClO) at 38 °C for 20 min and washed five times in sterile physiological saline (pH 7.4) by centrifugation at 500× *g* (5 min) at room temperature (22–24 °C). ***Step 2—Plate preparation for screening:*** Test and control compounds are dispensed, diluted in LB* into 384-well plates using a semi-automatic pipetting robot (ASSIST PLUS, Integra Biosciences) and 80 exsheathed L3s (xL3s) are added to individual wells. The compounds are arrayed on each plate, with 16 wells being negative-controls (with LB* + 0.4% DMSO) and four wells with each of the positive-control compounds: monepantel, moxidectin, monepantel+abamectin and M-666 (20 µM). ***Step 3—Measuring larval motility: ***Following a 90 h-incubation at 38 °C, 10% (*v*/*v*) CO_2_ and >95% of relative humidity, the motility of xL3s is measured using the WMicroTracker ONE instrument (Phylumtech, Argentina), and test-compounds with an anthelmintic effect (i.e., ‘hits’) are identified based on a ≥ 70% reduction in xL3 motility. ***Step 4—Microscopic examination:*** Following a total incubation period of 168 h (7 days) under the same incubation conditions, larval development and morphology (=phenotype) are assessed by light microscopy (100-times magnification).

Here, we established a whole-worm HTS assay in a 384-well plate format that is capable of screening ~10,000 compounds per week. This screening assay first assesses larval motility (at 90 h); after an extended incubation (168 h), developmental inhibition as well as changes in the phenotype of developing larvae are evaluated with respect to untreated controls (cf. Figure 6; steps 2 to 4). This platform is practical, quantitative and semi-automated, and overcomes the limitations of our previous 96-well plate assay [30], achieving a ≥ 10-times higher throughput and not requiring extensive technical skill to run or calibrate the assay. It costs about ~ USD 67,000 to set up; most of this expense relates to the purchase of a semi-automated liquid handling robot (at USD 30,000), two WMicroTracker ONE instruments (at USD 15,000 each) and a dissecting and a compound microscope (at USD 3000 each). In our laboratory, the established assay achieves a high signal-to-background ratio and a favourable and consistent Z’-factor (≥0.8), showing repeatable/reproducible results. Meticulous optimisation is critical, as is a sound understanding of the factors that can affect assay performance.

Clearly, standardisation of the number of worms per well and consistency in number among wells are crucial. Larval motility is recorded simultaneously in individual wells of the 384-well plate via infrared light beam-interference. The fluctuation in the signal resulting from larvae passing across and within the refraction of the infrared beam within a well is recorded as locomotion—referred to as ‘activity count’ [28]. Measuring the motility of ~ 80 xL3s within individual wells reduces experimental bias that can occur when lower or higher numbers of worms are used, and normalises the motility recorded in each well. The assay relies on the use of xL3s; large stocks of ensheathed L3s can be readily stored at 11 °C for periods of up to six months without an adverse effect on xL3 motility, which is a major advantage over other assays that rely on the use of stages, such as eggs or adult worms (harvested from experimentally infected animals) which cannot be stored like L3s.

Important is also an understanding of the technical aspects and settings of the WMicroTracker system. For *H. contortus* xL3s, we identified that different measurement modes (settings) had a profound effect on the measurement of motility (‘activity counts’). Empirically, we learned that Mode 1 in our assay *constantly* records all of the movement (activity counts) over time, and provides a representative quantitative measurement for individual wells (over a period of 15 min), whereas Mode 0 (default) records movement in a sliding time-window and normalises the data, leading to substantially lower ‘activity counts’. Clearly, there was a substantial difference in the performance of the assay, depending on the mode selected; this critical aspect might be overlooked when the WMicroTracker is used and has not been discussed previously in the published literature. Using Model 1, we achieved a high throughput in the assay by capturing larval motility within a short data acquisition period (15 min). We defined a critical measurement time-point, at which sufficient motility was measured without compromising the identification of ‘hit’ compounds. This contrasts with acquisition periods used in most published studies, which employed the WMicroTracker system for nematodes and in which motility was usually measured for periods of 3 h to 17.5 h [29,35,36,37,38], resulting in a considerably reduced throughput compared with the present assay protocol. The original ‘tracking’ system [28] was initially established for measuring *C. elegans* motility; the instrument was improved (WMicroTracker) and has since been applied to screen a range of other organisms including the nematodes *Cooperia oncophora*, *Ostertagia ostertagi*, *Teladorsagia circumcinta* and the zebrafish, *Danio rerio*, for alterations in motility [36,39], although some research groups might not have optimised it for the particular species under investigation. If optimised, this WMicroTracker-based platform should be applicable to a wide range of species of nematodes of animals (including humans), provided that the dimensions, motility and/or behaviour of the worms are suited to this system.

Using the xL3 motility threshold of 30% (Figure 4), we identified 42 ‘hit’ candidates; the expectation of such screens is that active compounds substantially reduce larval motility and/or development, eventually leading to worm morbidity and/or death (if compound effect is irreversible). However, consistent with the findings of Clare et al. [40], we observed that more than half (55%; 44,000 of 80,500) of the compounds screened here increased motility markedly beyond that of the negative-control (i.e., 101% to ~240%; see Figure 4). Although we do not yet have an explanation for this finding, we hypothesise that at least some of the compounds that induce increased motility (>100%) might ultimately lead to worm ‘burn-out’ and lethality over time, and, thus, might be candidates for further exploration. Although we do not yet have molecular biological insights into this ‘phenomenon’, we plan to explore some of the candidates that ‘excite’ the worms to exhaustion. To do this, we propose to undertake well-controlled, temporal in vitro*-*studies using xL3s and other developmental stages of *H. contortus*.

## 4. Materials and Methods

### 4.1. Procurement, Storage and Preparation of H. Contortus Larvae for Screening

*H. contortus* (Haecon-5 strain; ref. [41]) was maintained in experimental sheep as described previously [30,41] and in accordance with the institutional animal ethics guidelines (permit no. 1714374; The University of Melbourne). In brief, helminth-free Merino sheep (six months of age; male) were inoculated intra-ruminally with 7000 L3s of *H. contortus.* Four weeks after infection, faecal samples were collected each day, and L3s were produced from eggs by incubating faeces at 27 °C and >90% relative humidity for one week [30,41]. Then, L3s were sieved through two layers of nylon mesh (pore size: 20 µm; Rowe Scientific, Deveton, VIC, Australia) to remove debris or dead larvae, and stored at 11 °C for up to six months. Storage conditions for L3s were evaluated and standardised previously [30].

On the day of screening, L3s were exsheathed and sterilised by incubation in 0.15% (*v*/*v*) sodium hypochlorite (NaClO) at 38 °C for 20 min [42]. Following this incubation, exsheathed L3s (xL3s) were immediately washed five times in sterile saline by centrifugation at 500× *g* (5 min) at room temperature (22–24 °C). After the last wash, xL3s were immediately suspended in Luria-Bertani broth (LB: 10 g tryptone (cat. no. LP0042; Oxoid, Thermo Fisher Scientific, Waltham, MA, USA), 5 g yeast extract (cat. no. LP0042; Oxoid, USA), 5 g NaCl (cat. no. K43208004210; Merck, Kenilworth, NJ, USA) in 1 litre of reverse-osmosis deionised water). LB was autoclaved and supplemented with final concentrations of 100 IU/mL of penicillin, 100 µg/mL of streptomycin and 0.25 µg/mL of amphotericin B (Fungizone^®®^, cat. no. 15240-062, Gibco, Thermo Fisher Scientific, Waltham, MA, USA); this supplemented LB was designated LB*. The exsheathment and culturing procedures were standardised previously [30].

### 4.2. High Throughput Screening of Compounds for xL3 Motility Reduction at 90 h

The “Jump-stARter” library, containing 80,500 proprietary small molecular compounds, was provided by Janssen Pharmaceuticals Research and Development, Belgium via Johnson & Johnson (Dr Paul Jackson). These compounds were supplied at a concentration of 5 mM in 100% DMSO. Using a semi-automated liquid handling robot (VIAFLO ASSIST PLUS, Integra Biosciences, Zizers, Switzerland), compounds were individually diluted to 20 µM in LB* containing 0.4% (*v*/*v*) DMSO and subsequently dispensed in 20 µL into the wells of sterile 384-well flat bottom microtitre plates (cat. no. 3680; Corning, USA); 320 compounds were arrayed on each plate, with 16 wells being negative-controls (with LB* + 0.4% DMSO) and four wells containing each monepantel (Zolvix; Elanco, Greenfield, IN, USA), moxidectin (Cydectin; Virbac, Carros, France), monepantel/abamectin (Zolvix Plus; Elanco, USA) and compound MIPS-0018666 (abbreviated here as M-666; ref. [32]) as positive-controls (20 µM) (Figure 6). Following the dilution and dispensing of compounds into plates, 80 xL3s in 20 μL of LB* were transferred to individual wells (the optimisation of this number of larvae per well is described in subSection 2.2); after this step, the final concentrations were 20 µM of test- or positive-control compound; and 0.4% of DMSO. During dispensing, xL3s were maintained in a homogenous suspension using a constant stream of bubbles produced employing an aquarium pump (H2Pro, Melbourne, VIC, Australia). Plates were incubated in a water-jacketed CO_2_ incubator (Forma, model no. 311, Thermo Fisher Scientific, USA) at 38 °C, 10% (*v*/*v*) CO_2_ and a relative humidity of > 90%.

After 90 h of incubation of xL3s with compounds (20 μM), larval motility was measured for 15 min in individual wells of each plate by infrared light beam-interference [28] using a WMicroTracker ONE instrument (Phylumtech, Sunchales, Argentina). Raw data captured were normalised against measurements obtained for the positive (M-666) and negative (LB* + 0.4% DMSO) -controls to remove plate-to-plate variation by calculating the percentage of motility using the program GraphPad Prism v.9.1.0 (GraphPad Software, San Diego, CA, USA). A compound was considered active (‘hit’) if it reduced larval motility by ≥70%. To continually assess the performance of the screening assay, the Z’-factor [43] was calculated (using data for the DMSO and the M-666 controls) for individual plates (*n* = 251) and was consistently ≥0.8; reliable assays achieve a Z’-factor of between 0.5 and 1. The signal-to-background ratio (for the same controls) was consistently >200.

### 4.3. Assessing Larval Development and Phenotypes at 168 h

Following the measurement of xL3 motility (subSection 4.2), plates were incubated for a total period of 168 h in a humidified environment (water-jacketed CO_2_ incubator) at 38 °C and 10% (*v*/*v*) CO_2_. Then, worms were fixed with 40 µL of 1% iodine, and each well was examined at 60-times magnification (M80, Leica, Wetzlar, Germany) to assess larval development (based on the presence/absence of a well-developed pharynx; ref. [44]) and phenotype.

### 4.4. Dose-Response Assay

Dose–response relationships were established to estimate IC_50_ values for compounds against xL3s using an established method [30]. With reference to the two positive-control compounds (monepantel and moxidectin), IC_50_ values were established for hit compounds by two-fold serial dilution, starting at a concentration of 100 µM (18-points; in 50 μL of LB*; 100 µM to 0.76 nM), in 96-well plates (cat. no. 3596; Corning, Corning, NY, USA) with xL3s dispensed in 50 µL at a density of 300 per well [30]. After incubation at 38 °C and 10% (*v/v*) CO_2_ with >90% humidity, the motility of xL3s was measured at 90 h, and larval development at 168 h of incubation with compound. The compound concentrations were log_10_-transformed and fitted using a variable slope four-parameter equation, constraining the highest value to 100% using a least squares (ordinary) fit model employing GraphPad Prism software v.9.1.0. Compounds were tested in triplicate on three different days. IC_50_ were also established for other developmental stages, as required, using the same algorithm. For statistical analysis of larval motility, a one-way analysis of variance (ANOVA) with a Tukey’s multiple comparison test or an unpaired *t*-test was used to establish statistically significant differences.

## 5. Conclusions

The present HTS assay is practical and meets an industry standard, akin to that of Clare et al. [40], and is readily suited for use in effective public-private partnerships to discover new anthelmintic entities on a large scale. Our goal is to extend its use to a range of parasitic worms of economic importance (e.g., species of *Cooperia*, *Ostertagia* and *Trichostrongylus*) and human health significance—particularly those that cause neglected tropical diseases (NTDs), such as species of *Ancylostoma*, *Necator*, *Ascaris* and *Trichuris*. This latter focus is of particular relevance, given that the goals of the World Health Organization Roadmap for NTDs 2021–2030 are to develop more effective drugs and drug-combinations to improve patient outcomes, and to circumvent the ineffectiveness of some current anthelmintic drugs and possible drug resistance [45]. Currently, we are now in the process of screening collections of >200,000 synthetic and natural compounds, in collaboration with philanthropic partners, including the Medicines for Malaria Venture (MMV) and Griffith Institute for Drug Discovery (GRIDD).

## Data Availability

Data are within the article and Supplementary Materials.

## Supplementary Materials

The following are available online at https://www.mdpi.com/article/10.3390/ph14070616/s1, Figure S1: Anthelmintic activity of positive-control compounds in vitro against exsheathed third-stage larvae (xL3s) of *Haemonchus contortus*, Table S1: The 42 ‘hit’ compounds identified in the primary screen with in vitro-activity against exsheathed third-stage larvae (xL3s) of *Haemonchus contortus*, and Table S2: Distinct phenotypes of exsheathed third-stage larvae (xL3s) and fourth-stage larvae (L4s) of *Haemonchus contortus* observed in this study.
