# Supplementary material for: High-Throughput Phenotypic Assay to Screen for Anthelmintic Activity on Haemonchus contortus
Source: Pharmaceuticals (Basel). 2021 Jun 26;14(7):616. doi: 10.3390/ph14070616 (PMC8308562; doi:10.3390/ph14070616)
Supplement: Supplementary file 1 [file pharmaceuticals-14-00616-s001.zip › pharmaceuticals-1256235-supplementary.pdf]

Supplementary Materials

High-throughput phenotypic assay to screen for anthelmintic activity on *Haemonchus contortus*

Aya C. Taki [1], Joseph J. Byrne [1], Tao Wang [1], Brad E. Sleebs [1,2,3], Nghi Nguyen [2,3], Ross S. Hall [1], Pasi K. Korhonen [1], Bill C. H. Chang [1], Paul Jackson [4], Abdul Jabbar [1], Robin B. Gasser [1,*]

[1] *Department of Veterinary Biosciences, Melbourne Veterinary School, Faculty of Veterinary and Agricultural Sciences, The University of Melbourne, Parkville, Victoria 3010, Australia*
[2] *Chemical Biology Division, Walter and Eliza Hall Institute of Medical Research, Parkville, Victoria 3052, Australia*
[3] *Faculty of Medicine, Dentistry and Health Sciences, The University of Melbourne, Parkville, Victoria 3010, Australia*
[4] *Johnson & Johnson, Global Public Health, Janssen Research and Development, San Diego, California, USA*

---

* Corresponding author.
E-mail address: robinbg@unimelb.edu.au.

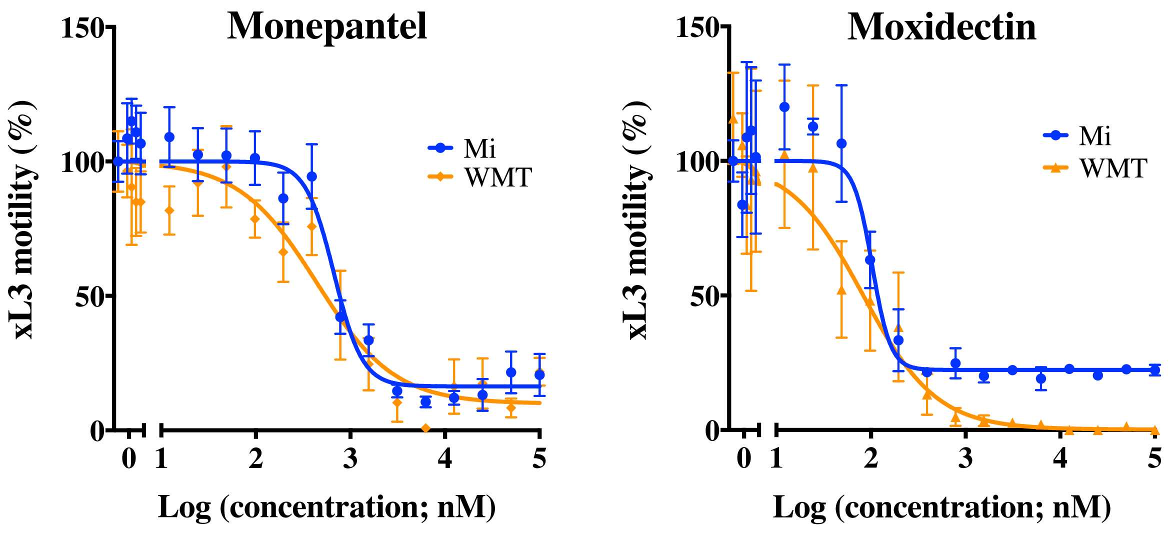

Figure. S1. Anthelmintic activity of positive-control compounds in vitro against exsheathed third-stage larvae (xL3s) of *Haemonchus contortus*. Larval motility values (WMT and Mi) were recorded using the WMicroTracker ONE instrument and the original screening assay [30], respectively, at 90 h after exposure to each of the compounds (monepantel and moxidectin). Data points are triplicates in three independent experiments on different days; the mean ± standard error of the mean (SEM).

Table S1. The 42 'hit' compounds identified in the primary screen with in vitro-activity against exsheathed third-stage larvae (xL3s) of *Haemonchus contortus*. Information about compounds (code, chemical name, SMILES and structure) and the percentage of motility reduction in xL3s at 90 h (primary screen) and the larval phenotypes (L4) detected at 168 h. A subset (*) of these 42 compounds was further assessed in dose-response assay (Fig. 5) and phenotypes recorded (cf. Table S2).

Compound code	Motility reduction (%) in xL3 at 90 h	Abnormal morphological phenotype detected at 168 h
UoM-7625	76.6	Not detected in the primary screen (nd)
UoM-2588	75.3	nd
UoM-9701	76.7	nd
UoM-8811*	81.4	'Evisceration' *(Evi)**
UoM-5416	71.3	nd
UoM-5470	70.5	nd
UoM-8574	83.5	nd
UoM-4502	71.0	nd
UoM-6793	72.9	'Coiled' *(Coi)*
UoM-2615	87.9	nd
UoM-6741	78.0	nd
UoM-4965	89.3	nd
UoM-0920	74.5	*Evi*
UoM-5126	73.4	nd
UoM-6404	88.8	nd
UoM-2807	81.4	nd
UoM-4068	74.4	nd
UoM-0377	73.7	nd
UoM-6038	70.5	nd
UoM-2808	82.9	nd
UoM-8496	83.5	nd
UoM-6088	78.6	*Evi*
UoM-4350	76.2	nd
UoM-9644	85.5	nd
UoM-9012	71.9	nd
UoM-7026	70.8	*Evi*
UoM-7300	72.8	nd
UoM-7024*	72.3	nd*
UoM-2533	72.0	nd
UoM-2866	77.4	nd
UoM-5961	90.5	nd
UoM-7347	85.8	nd
UoM-9466	79.3	nd
UoM-4380	79.3	nd
UoM-4423	79.2	nd
UoM-5812	70.8	*Evi*
UoM-8035*	90.5	nd*
UoM-3235	77.1	'Curved' *(Cur)*
UoM-7382	72.8	nd
UoM-4124	74.0	nd
UoM-4865	76.5	nd
UoM-0366	71.1	nd

* When subsequently tested individually in the dose-response assay (sub-section 3.4), compounds UoM-8811, UoM-7024 and UoM-8035 (highlighted in grey) markedly inhibited larval development, achieving IC_{50} values of ~ 4 µM, 25 µM and 41 µM, respectively (Fig. 5), with each of them inducing abnormal phenotypes (*Cur* and *Evi*) detected by detailed microscopic examination at 100-times magnification.

Table S2. Distinct phenotypes of exsheathed third-stage larvae (xL3s) or fourth-stage larvae (L4s) of *Haemonchus contortus* observed in this study.

Morphology*	Phenotype	Abbreviation	Description
Normal			
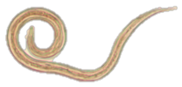	Wild-type (xL3)	WT	Larvae with 'normal' morphology and motility.
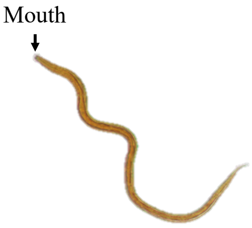	Wild-type (L4)	WT	Larvae with 'normal' morphology and motility. A buccal capsule (mouth) and pharynx present (cf. [45]).
Abnormal			
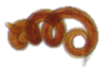	Coiled	*Coi*	Tightly coiled and not motile.
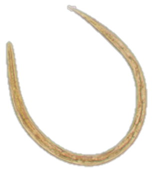	Curved	*Cur*	U-shape and not motile.
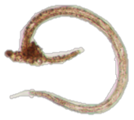	Evisceration	*Evi*	Evisceration through the excretory pore of the worm (anterior); not motile. Exhibited by L3s or premature L4s (sheathed); lethal.

* The lengths of wild-type xL3s and L4s cultured *in vitro* in LB* medium (containing 0.4 % DMSO) for 90 h and 168 h are ~ 600-650 µm and 700-750 µm, respectively.
